# An Assessment of the *Lolium perenne* (Perennial Ryegrass) Seedborne Microbiome across Cultivars, Time, and Biogeography: Implications for Microbiome Breeding

**DOI:** 10.3390/microorganisms9061205

**Published:** 2021-06-02

**Authors:** Ian Tannenbaum, Brendan Rodoni, German Spangenberg, Ross Mann, Tim Sawbridge

**Affiliations:** 1 Agriculture Victoria, AgriBio Centre for AgriBioscience, Bundoora, VIC 3083, Australia; brendan.rodoni@agriculture.vic.gov.au (B.R.); german.spangenberg@agriculture.vic.gov.au (G.S.); ross.mann@agriculture.vic.gov.au (R.M.); tim.sawbridge@agriculture.vic.gov.au (T.S.); 2 School of Applied Systems Biology, La Trobe University, Bundoora, VIC 3083, Australia

**Keywords:** 16S rRNA, biogeography, endophytes, *Lolium perenne*, microbial diversity, microbiome, metagenomics, seed

## Abstract

Research into the bacterial component of the seed microbiome has been intensifying, with the aim of understanding its structure and potential for exploitation. We previously studied the intergenerational seed microbiome of one cultivar of perennial ryegrass with and without one strain of the commercially deployed fungal endophyte *Epichloë festucae* var. *lolii*. The work described here expands on our previous study by exploring the bacterial seed microbiome of different commercial cultivar/*Epichloë festucae* var. *lolii* combinations in collections of single seeds from the harvest year 2016. In this dataset, a cultivar effect could be seen between the seed microbiomes from cultivars Alto and Trojan. The bacterial component of the seed microbiome from pooled seeds from a single cultivar/*E*. *festucae* var. *lolii* combination harvested from 13 seed production farms around Canterbury in the year 2018 was also studied. This dataset allows the effect of different production locations on the bacterial seed microbiome to be examined. By comparing the two sets of data, bacteria from the genera *Pantoea*, *Pseudomonas*, *Duganella*, *Massilia*, and an unknown Enterobacteriaceae were observed to be in common. This core bacterial microbiome was stable over time but could be affected by supplemental taxa derived from the growth environment of the parental plant; differing microbiomes were seen between different seed production farms. By comparison to a collection of bacterial isolates, we demonstrated that many of the members of the core microbiome were culturable. This allows for the possibility of exploiting these microbes in the future.

## 1. Introduction

Temperate pastures in Australia and New Zealand commonly grow perennial ryegrass (*Lolium perenne*) as feed for livestock, particularly beef and dairy cattle. Among the many species of pasture grasses in use, perennial ryegrass is favoured for its persistence and feed quality. Some of the persistence of perennial ryegrass has been attributed to the symbiotic relationship of the grass to a resident fungal endophyte, *Epichloë festucae* var. *lolii* [1,2]. Many different combinations of fungal endophyte genotypes and perennial ryegrass cultivars are commercially available, each of which provides differing pasture performance, allowing farmers to tailor combinations depending on their needs.

While there has been a considerable amount of research dedicated to the effects of *Epichloë* species on pastures, there has been little dedicated to other specific fungi or bacteria, or the wider microbiome. The bacterial microbiome of plants vary based on the host environment (e.g., rhizosphere, phyllosphere, endosphere) with each containing core phyla [3,4]. What research that has been done on the pasture microbiome has often been in the context of *Epichloë* fungal endophytes. Recently, Nissinen et al. [5] demonstrated in the leaves of four-month-old tall fescue that resident fungal endophytes bore little effect on the host bacterial microbiome; however, Tannenbaum et al. [6] identified an effect of *Epichloë festucae* var. *lolii* on the bacterial microbiome in pooled young perennial ryegrass seedlings of a single cultivar. 

Several plants have been examined to determine their seed microbiome structure, including rice, barley, field pumpkin, and rapeseed [7,8,9,10]. These previous studies demonstrated the value of seed microbiomes, in particular, the value of exploiting the indigenous bacteria to improve breeding strategies and plant resistance to pathogens. Hence, understanding the complexities and determining factors of seed microbiomes offers insights into their function during, prior, and after germination. 

One factor which can define the plant microbiome is plant genotype. Shenton et al. [11] observed a weak genotype effect on the bacterial microbiome of *Oryza sp.* where there was a shift in the relative abundance of some methanotrophs in the rhizosphere. As *L*. *perenne* is self-incompatible and not able to self-pollinate, all single plants and seeds represent single genotypes [12]. As such, there may be differences between the microbiome of single seeds of self-incompatible plant species due to seed genotype. Of the available research, temporal effects have also been observed by Wagner et al. [13] on the leaf and root microbiome of *Boechera stricta* (*Brassicaceae*) when harvested from the same location from different years. However, one of the greatest deterministic factors of the microbiome composition was geographic distance and edaphic conditions [14]. Fan et al. [14] showed that the physical properties of soil from different geographic locations affected the microbiome community structures. Be it barley [15] or soybean [16], different biogeographical locations are subject to vast arrays of climatic conditions, such as precipitation, temperature, pH, and light [17]. Such climatic effects influence microbiome community structures, as shown by Bryant et al. [18] whereby altering the moisture content and pH of soils resulted in differing proportions of Acidobacteria. 

In this study, we assessed the seed microbiome of two *L. perenne* cultivars, Trojan and Alto. The effect of biogeography was studied using the Trojan cultivar with the same fungal endophyte combinations across 13 seed production farms (harvest year 2018). This can aid in the understanding of the effect of commercial breeding strategies on seedborne microbiomes. The effect of cultivar was also studied in Trojan and Alto (harvest year 2015–2016), at a single seed level, to examine variation within a single seed and between cultivars. We also assessed the impact of fungal endophyte loss within the microbiome of *E*. *festucae* var. *lolii* containing batches as commercial seed batches require >80% infection rate. Finally, a temporal aspect was studied to assess the stability of the core microbiome across varying harvest years. 

## 2. Materials and Methods 

### 2.1. Experimental Design

Following our prior study, we sought to elucidate additional factors and their influence on the bacterial microbiome of *L*. *perenne* [6]. Here, we assessed the microbiome profiles of multiple cultivar/*E. festucae* var. *lolii* combinations between two cultivars (Alto and Trojan).

To determine inter-seed microbiome variation and effect of fungal endophyte, five seed batches of either *L. perenne* cv. Trojan or Alto containing the fungal endophytes AR1, AR37, SE or without fungal endophyte (WE), were harvested in 2015–2016 by Barenbrug Agriseeds New Zealand (Table 1); hereon referred to as Single Seeds or singularly as the combination specified in Table 1 (e.g., AltoAR37). As commercial seed batches require >80% infection rate of *E*. *festucae* var. *lolii*, Single Seeds were tested for the presence/absence of viable fungal endophyte to determine the effect of fungal endophyte loss or drop-off on the microbiome within a seed batch.

**Figure 1:**
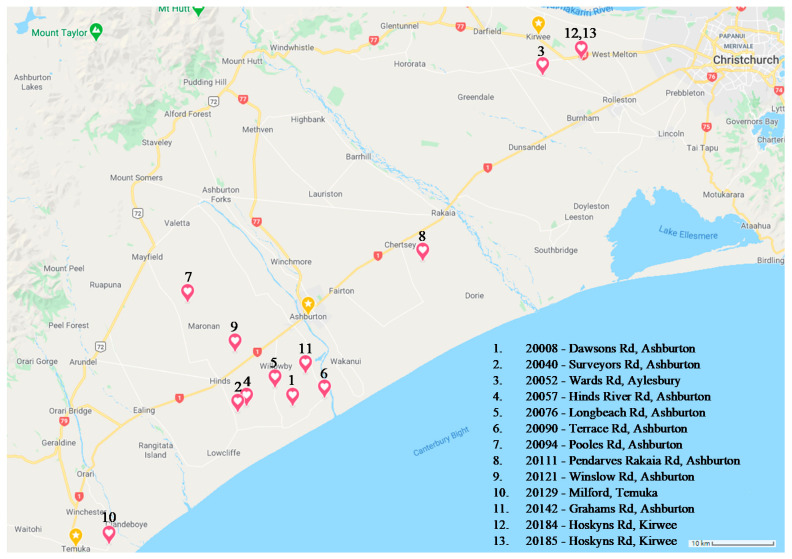
Approximate biogeographical locations of each Biogeography Seed accession.

To determine effect of biogeography on the microbiome, 13 seed batches of *Lolium perenne* cv. Trojan containing a mixed consortium of fungal endophytes were harvested in 2018 by Barenbrug Agriseeds New Zealand from multiple farms across Ashburton, Kirwee, Aylesbury, and Temuka, New Zealand (Table 2); hereon referred to collectively as the Biogeography Seed or singularly as per the source numbers described in Table 2. The approximate geographical origin of these accessions is available in the (Figure 1). 

### 2.2. Perennial Ryegrass Seed Processing

All seeds were surface-sterilised as per Kaur et al. [2] with the following modifications; the H_2_SO_4_ step was removed, seeds were washed in 3% NaClO for 3 min and five times in sterile Milli-Q H_2_O for 1 min. All seeds were germinated on moistened filter paper in sterile Petri dishes and incubated in the dark, at room temperature, for 7 days. Seedlings of similar size were harvested.

### 2.3. DNA Extraction and 16S Amplicon Sequencing

For the microbiome profiling of Single Seeds, single roots and shoots were collected from seedlings from each cultivar/*E*. *festucae* var. *lolii* combination (Table 1). For the microbiome profiling of Biogeography Seeds, 25–50 roots were collected from seedlings from each source. Roots were pooled into sets of 5, creating 5–10 replicates representing each source. DNA extraction was performed for all Single Seed and Biogeography Seed samples using the QIAGEN MagAttract 96 DNA Plant Core Kit according to manufacturers’ instructions with minor modifications for use with a Biomek FX liquid handling station.

From the Single Seeds, 44 shoots from Alto AR1, Alto AR37, and Trojan AR1 and 22 shoots from each of Alto SE and Alto WE were assessed for fungal endophyte presence/absence using a “kompetitive” allele-specific (KASP) assay (Supplementary Method S1) [19]. Two to six roots per KASP-verified cultivar/*E*. *festucae* var. *lolii* combination were kept individually and prepared for DNA extraction. The bacterial microbiome was profiled targeting the V4 region of the 16S rRNA gene according to the Illumina 16S Metagenomic Sequencing Library Preparation protocol (Supplementary Method S2), with minor modifications to include the use of peptide nucleic acid (PNA) PCR blockers to reduce the amplification of 16S rRNA gene sequences derived from the plant organelle genome [13,20]. Paired-end sequencing was performed on an Illumina HiSeq3000 using 2 × 150 bp. All Illumina sequences have been submitted to the NCBI Short Read Archive (SRA Accession PRJNA577475).

### 2.4. Data Processing and Statistical Analysis

Sequence data were processed using QIIME2 as per Tannenbaum et al. [6] with modification; reads were rarefied to 7000. Dominant OTUs were defined as being an OTU that represented >1% of the total read abundance in any one sample. The QIIME2 generated OTU tables were post-processed to generate the Dominant OTU table. These OTUs were then ranked by their proportion abundance (Supplementary Information 2). To assess the culturability of the most dominant ranked taxa (Rank 1), the 16S V4 tags were mapped to the full 16S sequences of known bacterial isolates from *L*. *perenne* using NINJA-OPS [6,21].

## 3. Results

To examine the variability in seed microbiomes of perennial ryegrass, two sets of seeds were examined. Set one comprised individual seedlings derived from surface sterilised seeds batches obtained in 2015–16 from Barenbrug Agriseeds New Zealand. These comprised 5 batches encompassing 2 different cultivars containing different *E*. *festucae* var. *lolii* endophyte strains (Table 1). To examine any consistent effect of the fungal endophyte at a single seed level, the DNA extracted from the seedlings was assessed with a KASP assay and the DNA classified as endophyte containing or endophyte-free. Of the screened shoots, the correct fungal endophyte was detected in Alto AR1 (25/44), Alto AR37 (35/44), Trojan AR1 (13/44), Alto SE (19/22), and absence thereof in Alto WE (20/22). The second set of seeds was derived from batches comprising one commercial cultivar/*Epichloë festucae* var. *lolii* combination obtained in 2018 from Barenbrug Agriseeds New Zealand from 13 different seed production farms (Table 2; Figure 1). Commercial seed/*E*. *festucae* var. *lolii* combinations are required to contain fungal endophyte in at least 80% of the seed, so KASP assays were not performed. For suitable microbiome coverage and to reduce the effect of the resident fungal endophytes, five seedlings were pooled for DNA extraction.

### 3.1. Variation of Single Seed Microbiomes Between Cultivar/E. Festucae var. lolii Combinations

**Figure 2:**
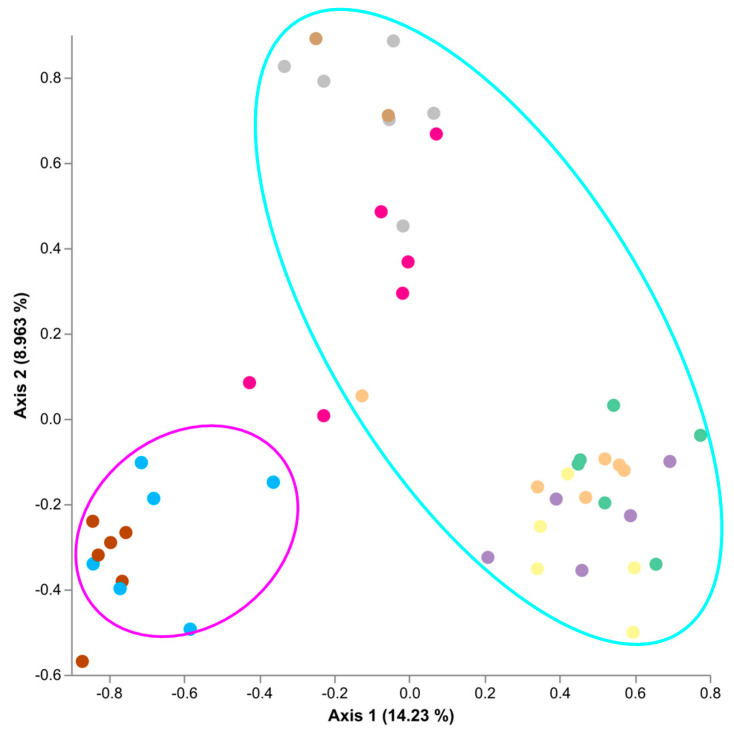
Jaccard similarity plots demonstrating the differences between Single Seeds; Trojan (cyan) and Alto (magenta) rings.

To assess the effect of cultivar, *E*. *festucae* var. *lolii* strains, and fungal endophyte presence/absence on the Single Seed microbiome, a qualitative Jaccard similarity analysis was conducted using the q2-diversity module of QIIME2 (Figure 2). Primary separation was observed between cultivar accounting for 14.23% of the microbiome variability across Axis 1. Secondary separation was observed between AR fungal endophytes and SE accounting for 8.963% of the microbiome variability along Axis 2. Alto WE had the least impact of the microbiome variability along Axis 2. There was no clear separation of replicates which were positive for their respective fungal endophyte and those which tested negative for their respective fungal endophyte.

**Figure 3:**
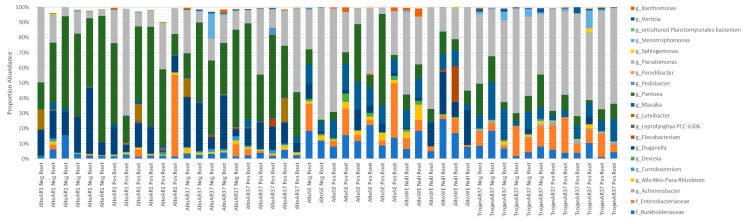
A comparison of genus level profiles of 41 dominant OTUs that each accounted for >1% of the total number of reads from Single Seeds.

There was conservation of 8 of 20 ( >50% of replicates) genera between Single Seed replicates (Figure 3). Despite the conservation, there was considerable variance of their relative abundance (Figure 3). Of the conserved genera, the most dominant were *Pantoea*, *Duganella*, and *Pseudomonas*, accounting for >60% of the microbiome in 37 of 48 Single Seed replicates.

A cultivar effect was observed in Single Seeds when comparing Alto and Trojan cultivars in combination with AR37 fungal endophyte. Alto AR37 had a greater association to *Pantoea* (15.52–63.39%) and *Duganella* (4.56–35.08%) than Trojan AR37 (2.10–21.65% and 0.0–5.13%, respectively) (Figure 3). Trojan AR37 had a greater association to *Pseudomonas* (30.7–68.7%) and an Enterobacteriaceae (1.97–23.11%) than Alto AR37 (7.28–55.31% and 0.0–7.87%, respectively) (Figure 3).

Effects of fungal endophytes were observed when considering only the Alto cultivar. Alto seed containing AR1 and AR37 fungal endophytes yielded similar bacterial profiles whereas SE and WE shared similar profiles (Figure 3). SE and WE had greater associations to *Massilia* (1.18–18.25%), Allo-Neo-Para-Rhizobium (0.0–10.22%), and members of families Enterobacteriaceae (0.21–35.82%) and Burkholderiaceae (5.09–26.2%) which were unresolved to genus than AR fungal endophyte containing seeds (0.12–5.04%, 0.01–3.49%, 0.0–7.87% with an occurrence at 53.61% and 0.95–15.54%, respectively) (Figure 3). When considering only Alto SE and WE, Alto SE had greater associations to *Pseudomonas* (3.89–73.39%), *Pantoea* (1.04–60.37%), and a member of the family Enterobacteriaceae (0.21–35.82%) which was unresolved to genus than Alto WE (15.21–66.9%, 7.03–14.36%, and 1.17–9.78%, respectively) (Figure 3). Alto WE had greater associations to *Duganella* (1.02–27.76%), *Flavobacterium* (0.0–23.5%), and *Xanthomonas* (0.0–5.49%) than Alto SE (0.2–13.4%, 0.0–0.0%, and 0.01–2.8%, respectively) (Figure 3). There were no observable differences between replicates which were positive for their respective fungal endophyte and those which tested negative for their respective fungal endophyte.

### 3.2. Biogeography Impacts the Lolium Perenne Seedborne Microbiome

**Figure 4:**
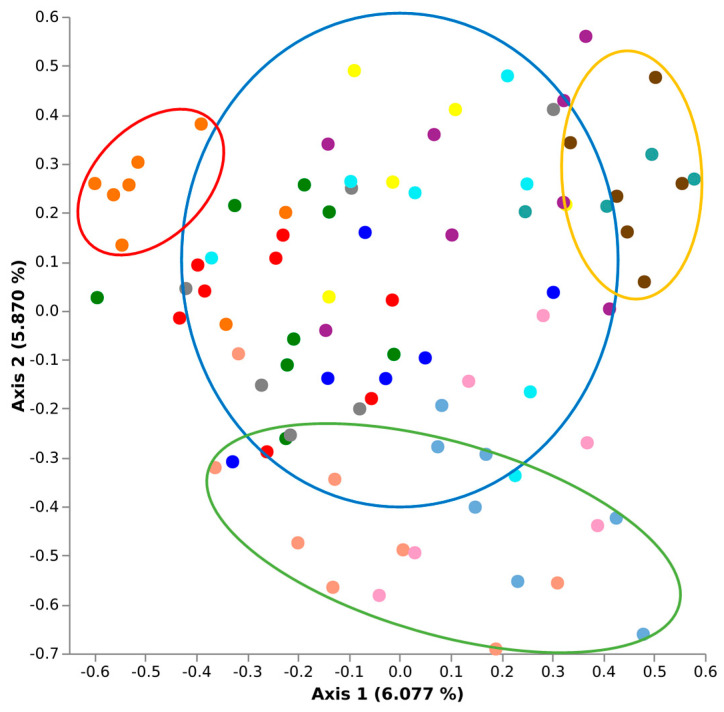
Jaccard similarity plots demonstrating a geographical effect on the Biogeography Seed microbiomes; Ashburton (central, blue), Aylesbury (north, red), Temuka (south, orange), and Kirwee (north, green) rings.

To assess the effects of biogeographical distance on the Biogeography Seed microbiome, a qualitative Jaccard similarity analysis was conducted using the q2-diversity module of QIIME2 (Figure 4). When comparing all Biogeography Seed, primary separation was observed between north (excluding Kirwee), central, and south regions, accounting for 6.608% of the microbiome variability along axis 1. A secondary separation was observed which could not be attributed to geographical distance, accounting for 5.487% of the microbiome variability along axis 2. The secondary separation that was observed was the Kirwee farms separating for all other farms.

**Figure 5:**
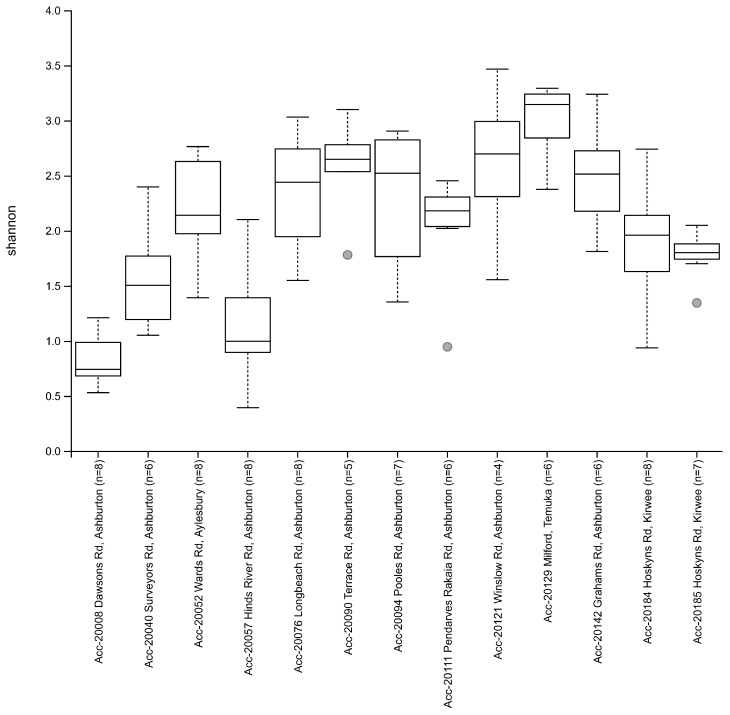
Estimated Shannon alpha diversity indexes between Biogeography Seed.

The Shannon’s alpha diversity median ($\tilde{x}$) value for the Biogeography Seed accessions were categorised into three tiers; low ($\tilde{x}$ ≤ 1.50), moderate (1.50 < $\tilde{x}$ < 3.00), and high ($\tilde{x}$ ≥ 3.00). Low alpha diversity (<1.05) was observed in Biogeography Seed accessions 20008 (Dawsons Rd), 20057 (Hinds River Rd), and 20040 (Surveyors Rd), which were in close proximity to each other within the central Ashburton region (Figure 5). Moderate Shannons’s diversity (1.78–2.70) was observed in all other accessions except for 20129 (Milford). High alpha diversity (3.15) was observed in accession 20129 (Milford), which was the southernmost assessed farm.

### 3.3. Microbiome Variation between Seed Production Farms

**Figure 6:**
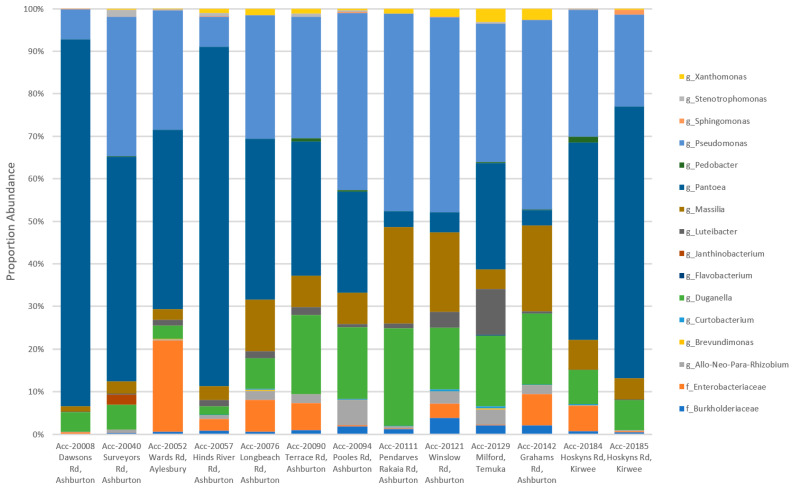
A comparison of genus level profiles of 39 OTUs that each accounted for >1% of the total number of reads from the Biogeography Seed.

A total of 16 genera were detected representing 39 OTUs, two of which remained unresolved at the family level. When the OTU level identifications were summarised to the genus level, more observable consistencies were seen between Biogeography Seed accessions (Figure 6).

Biogeography Seed accessions 20008 (Dawsons Rd), 20040 (Surveyors Rd), and 20057 (Hinds River Rd) in the low-diversity category were dominated by *Pantoea* (52.58–86.02%) and were in close proximity. Accession 20129 (Milford) in the high-diversity category had the greatest proportion of *Luteibacter* (10.54%) and *Flavobacterium* (0.23%) and was the southernmost assessed farm. Accession 20052 (Wards Rd) in the moderate-diversity category, with the exception of two single replicates from accessions 20076 (Longbeach Rd) and 20121 (Winslow Rd), had the greatest proportion of an unknown Enterobacteriaceae (21.38%) and was the northernmost assessed farm (excluding Kirwee). The remaining accessions in the moderate category had variable microbiomes where the variation could not be attributed to geographic distance.

### 3.4. The Temporal Effect on Seed Microbiomes

**Figure 7:**
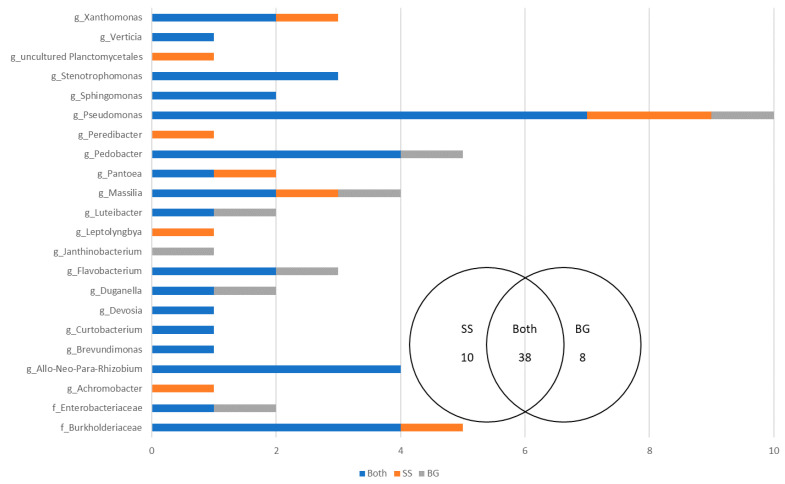
The number of shared and supplemental OTUs between the Single Seeds (SS)—2015–2016 and the Biogeography Seed (BG)—2018.

To assess the effect harvest year on perennial ryegrass seed microbiomes, a comparison was made between Single Seeds and Biogeography Seeds which were harvested in 2015–2016 and 2018, respectively. The analysis focused on the dominant microbes (>1% of reads), which accounted for 56 OTUs and represented 22 genera (Figure 7). A total of 38 OTUs were shared between the two harvest years indicating that a large proportion of the OTUs present in the seed microbiome were common across time. There were 18 OTUs that were unique to either the Single Seed harvest year (2015–2016, 10 OTUs) or the Biogeography Seed harvest year (2018, 8 OTUs). There were 17 genera that were always present in seed microbiomes, irrespective of the harvest year, including *Stenotrophomonas*, *Sphingomonas*, *Devosia*, *Curtobacterium*, *Brevundimonas*, and Allo-Neo-Para-Rhizobium. There were 4 genera that were exclusive to the 2015–2016 harvest year, including an uncultured Planctomycetales, *Peredibacter*, *Leptolyngbya*, and *Achromobacter*. There was one genus that was exclusive to the 2018 harvest year, *Janthinobacterium*. The *Pseudomonas* genus had the most OTUs (10), with 7 being shared across the two harvest years.

**Figure 8:**
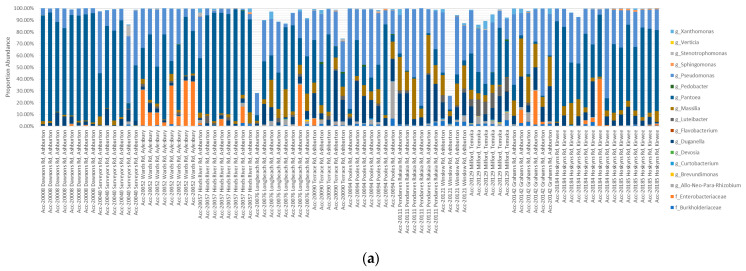

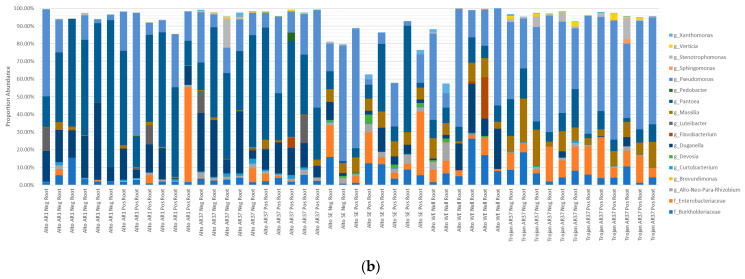
Comparison of the contribution of the 38 shared OTUs from Biogeography Seeds (**a**) and from Single Seeds (**b**).

The percentage abundance of OTUs that were shared ranged from 57.49–99.94% of the Single Seed (2015–2016) microbiome and 25.78–99.94% of the Biogeography Seed (2018) microbiome, with an average of 92.56% and 96.12%, respectively (Figure 8). In contrast, the percentage abundance of OTUs that were supplemental ranged from 0–41.6% for the Single Seed (2015–2016) and 0–72.99% for the Biogeography Seed (2018), with an average of 3.41% and 6.16%, respectively (Supplementary Figure S1). The genera with the highest average relative abundance were *Pantoea*, *Pseudomonas*, Enterobacteriaceae, and *Duganella*, comprising up to 94.93%, 84.89%, 53.6%, and 43.54% of the microbiome, and were always shared across the two harvest years.

To further ascertain the most important OTUs across the two harvest years, an analysis was undertaken to identify the OTUs with the highest abundance in any one replicate (Rank 1 taxa). Fourteen of the 56 OTUs met this criterion and represented the genera *Pantoea*, *Pseudomonas*, *Duganella*, *Massilia*, *Flavobacterium*, Allo-Neo-Para-Rhizobium, and an unknown genus from Enterobacteriaceae and Burkholderiaceae (Table 3). Of these OTUs, *Pantoea* has the highest relative abundance in 49% of replicates, followed by a *Pseudomonas* OTU in 7% of replicates.

### 3.5. Culturability of the Dominant Microbiome

To determine the culturability of the Rank 1 taxa, the V4 tags of the 14 Rank 1 taxa were mapped to known isolates. Twelve of the 14 Rank 1 OTUs aligned to four known isolates at 100% homology and to six known isolates at 97% homology (Table 3) [6]. OTUs 1–5, 10, and 12 corresponded to the genera *Pantoea*, *Duganella*, *Pseudomonas*, Allo-Neo-Para-Rhizobium, *Massilia*, and two additional species from *Pseudomonas* respectively were present in >50% of replicates (Any Rank) in harvest year 2018 (Biogeography Seed) (Table 3). OTUs 1–8 corresponded to the genera *Pantoea*, *Duganella*, *Pseudomonas*, Allo-Neo-Para-Rhizobium, *Massilia*, an unknown species from Enterobacteriaceae, and two additional species from *Pseudomonas* respectively were present in >50% of replicates (Any Rank) in the harvest year 2015–2016 (Single Seed) (Table 3). OTUs 1–5 corresponded to the genera *Pantoea*, *Duganella*, *Pseuodomonas*, Allo-Neo-Para-Rhizobium, *Massilia* and were present in >50% of replicates in both harvest years, suggesting a core microbiome to *L*. *perenne*.

## 4. Discussion

### 4.1. The Core and Supplemental Microbiome

A core microbiome was detected within each of the assessed batches (Single Seed and Biogeography Seed) of perennial ryegrass seed. The genera *Pantoea*, *Pseudomonas*, *Duganella*, *Massilia*, and an unknown Enterobacteriaceae were persistent as Rank 1 taxa and detected across all assessed Biogeography Seed and Single Seed batches. This is reflective of previous descriptions of broader Poaceae plant microbiomes including the seed of *L. perenne* [6] and components of sugarcane [22]. Of these dominant taxa, an OTU from the genus *Pantoea* was observed to be the most common Rank 1 taxa and had complete persistence within both the 2015–2016 (Single Seed) and 2018 (Biogeography Seed) harvest year batches. This indicated a persistent and stable core microbiome in *L. perenne*. The remainder of the microbiome was more transient and representative of the seedling genotype, cultivar, and/or growth environment.

The proportional abundance of supplemental taxa was very low when compared to the core taxa. This indicates that the supplemental taxa are not able to dominate a sample to the same extent as a taxon that was core by the two batches, i.e., a more common taxon. This suggests a niche that can be filled by supplemental bacteria depending on the surrounding environment. For example, a new taxon of Burkholderiaceae is seen in the Trojan AR37 Single Seeds, and a *Janthinobacterium* is seen in samples from Surveyors Road.

Resident fungal endophytes of *Lolium* spp. have variable alkaloid profiles which can alter the conditions in the hosts’ internal tissue and seed microbiomes [6,23]. The presence/absence of a viable fungal endophyte did not appear to have an impact on the microbiome profile within a batch of the endophyte-containing seed. Single Seed microbiomes with a detectable fungal load were comparable to those with no detectable fungal endophyte. This suggests the microbiome is maintained from the mother plant containing a resident fungal endophyte before its loss in the germinating seedling.

### 4.2. Biogeography Drives the Composition of the Biogeography Seed Microbiome

There was a clear effect of biogeography on the composition of the Biogeography Seed microbiome and appeared to correspond to latitudinal geographic position in most cases. For instance, farms in the northern region of Canterbury clustered together, farms in the central region of Canterbury clustered together, and farms in the southern region of Canterbury clustered together. A recent study which addressed biogeography on the microbiome of *Agave* described it as a driving factor of community composition [24]. While biogeography is a key factor in defining the microbiome, there are also other likely variables at play, including climatic and edaphic factors. A study of root-associated bacteria in soybean across China demonstrated that edaphic factors had greater effects on the bacterial community than climatic factors or geographical origins [16]. The Canterbury region, although a relatively small region compared to the above mentioned studies which assessed large spans of land up to 800,000 km^2^ [14,16], still has varying soil types, microclimates, pH, nutrient availability, and cultural practices that can affect community composition and structure. These factors may have contributed to the assembly of the seed microbiome from farms around Kirwee in the northern region of Canterbury that did not cluster according to latitudinal geography.

Although biogeography was determined to be a key factor, there was a set of taxa that was common throughout all assessed locations. Shade et al. [25] hypothesised that the seed microbiome reflects a key component of the parental plants’ microbial consortia that can be used as a starting point for the establishment of a new microbiome assemblage in a daughter plant. While each of the Biogeography Seed accessions originated from the same parental seed bulk mixture to make the Trojan cultivar, 11 genera were conserved when grown in different locations. Many of the taxa identified across the Biogeography Seed were also identified as Rank 1 taxa in the Single Seed microbiomes. The continuation of the prominent genera suggests the importance of the seedborne consortia in the viability of the seed for early development and germination [26].

### 4.3. Microbiome Breeding

The Nei’s genetic distance between cultivars Alto and Trojan has been measured between 0.0069–0.0078 and up to 0.0274 to other perennial ryegrass cultivars [12]. This shows that Alto and Trojan are genetically similar and could account for the observed similarities between their microbiomes. Genetic distance also exists within cultivars as *L*. *perenne* does not self-pollenate. As such, all individual plants have different genotypes [27,28]. In this study, a minor seed effect was observed between Single Seed microbiomes. The effect of genotype on the seed microbiome within a plant species remains largely unstudied. The effect of genotype has been evaluated on the rhizosphere microbiome, and in the case of *Oryza* species/genotypes, little effect was seen on the microbial composition [11]. In contrast, there has been an observable genotype effect in the root/rhizosphere microbiome of *Zea mays*, *Triticum aestivum*, and *Picea sp.* [29,30,31].

Each of the Rank 1 OTUs had shown a capacity to dominate the seed microbiome. An OTU from genus *Pantoea* was the most likely to dominate the microbiome. Species with the capacity to dominate seed microbiomes would be preferential candidates for further study with the potential to adjust or customise core taxa. Past studies suggest that some taxa are selected for functions which they provide to the host. When considered as a holobiont, perennial ryegrass and its close association to the observed core taxa could be an evolutionary product [32]. Strong preferences on microbial selection or conservation opens doors to microbiome modifications; replacement of core taxa using closely related species or the addition of supplementary taxa as it will unlikely dominate the stable core species. Many of the observed core taxa have been previously cultured (Table 3). This could allow for the formation of bacterial endophyte banks from which bacterial taxa could be selected for inoculates. Taxa with low rates of dominance may also be exploitable across a batch of seed to apply pasture-wide traits.

In this study, we found that some taxa, particularly those from the genera mentioned above dominated the seed microbiome of *L*. *perenne* more frequently than others. Due to their prevalence and apparent stability in the microbiome, these taxa could form an initial collection for prospective members to be included in artificial seed microbiomes. Some close relatives of the dominant core taxa have been previously characterised with a variety of beneficial effect, including nitrogen fixation, indole-3-acetic acid production, and biological effects towards other bacteria and fungi [33,34,35,36]. Building seed inoculates around highly functional taxa closely related to the native constituents may offset and replace them during plant maturation. The research presented here may assist in the further study of microbiome customisation in agriculture and more directed microbial manipulation in seed production.

## Figures and Tables

**Table 1:** Cultivar/*E*. *festucae* var. *lolii* combinations of *L. perenne* Single Seed accessions.

Cultivar	Fungal Endophyte	KASP	Number of Seedlings
Alto	AR1	+	6
		−	6
	AR37	+	5
		−	5
	SE	+	6
		−	2
	WE	NA	6
Trojan	AR37	+	6
		−	6

**Table 2:** The biogeographical locations of Biogeography Seed accessions.

Species	Cultivar	Source	Location
*Lolium perenne*	Trojan	20008	Dawsons Rd, Ashburton
		20040	Surveyors Rd, Ashburton
		20052	Wards Rd, Aylesbury
		20057	Hinds River Rd, Ashburton
		20076	Longbeach Rd, Ashburton
		20090	Terrace Rd, Ashburton
		20094	Pooles Rd, Ashburton
		20111	Pendarves Rakaia Rd, Ashburton
		20121	Winslow Rd, Ashburton
		20129	Milford, Temuka
		20142	Grahams Rd, Ashburton
		20184	Hoskyns Rd, Kirwee
		20185	Hoskyns Rd, Kirwee

**Table 3:** Mapping of the 14 Rank 1 OTUs against isolates obtained from *Lolium perenne*.

		Homology to Isolates		Any Rank (% of Reps)		Rank 1 (% of Reps)	
OTU	Name	97%	100%	NZ	SS	NZ	SS
OTU_1	g_Pantoea	*Erwinia persicina*		100%	100%	54%	40%
OTU_2	g_Duganella			99%	85%	6%	6%
OTU_3	g_Pseudomonas_6	*Pseudomonas poae*	*Pseudomonas poae*	51%	81%	2%	11%
OTU_4	g_Allo-Neo-Para-Rhizobium	*Rhizobium skierniewicense*	*Rhizobium skierniewicense*	91%	79%	1%	0%
OTU_5	g_Massilia	*Massilia aurea*		99%	77%	3%	2%
OTU_6	f_Enterobacteriaceae_1	*Erwinia persicina*	*Erwinia persicina*	43%	75%	6%	6%
OTU_7	g_Pseudomonas_5	*Pseudomonas cichorii*	*Pseudomonas cichorii*	0%	58%	0%	4%
OTU_8	g_Pseudomonas_2	*Pseudomonas cichorii*		3%	52%	1%	17%
OTU_9	f_Burkholderiaceae	*Acidovorax lacteus*		0%	48%	0%	2%
OTU_10	g_Pseudomonas_4	*Pseudomonas cichorii*		100%	40%	8%	0%
OTU_11	g_Flavobacterium			13%	38%	0%	2%
OTU_12	g_Pseudomonas_1	*Pseudomonas cichorii*		85%	33%	11%	0%
OTU_13	g_Pseudomonas_3	*Pseudomonas poae*		25%	29%	3%	9%
OTU_14	f_Enterobacteriaceae_2	*Erwinia persicina*		22%	0%	3%	0%

Identification made using full length 16S rRNA and is closely related to *Pantoea*.

## Data Availability

All Illumina sequences have been submitted to the NCBI Short Read Archive (SRA Accession PRJNA577475). The resulting OTU table are available in Supplementary Information 2.

## Supplementary Materials

The following are available online at https://www.mdpi.com/article/10.3390/microorganisms9061205/s1, Method S1: Endophyte Identification and Viability (KASP Assay), Method S2: Illumina library preparation, Method S3: Figure S1: Comparison of the contribution of the 8 unique dominant taxa from Biogeography Seed and 10 unique dominant taxa from Single Seed; Abundance and Ranked OTU Tables.
