# Supplementary material for: An Assessment of the Lolium perenne (Perennial Ryegrass) Seedborne Microbiome across Cultivars, Time, and Biogeography: Implications for Microbiome Breeding
Source: Microorganisms. 2021 Jun 2;9(6):1205. doi: 10.3390/microorganisms9061205 (PMC8228030; doi:10.3390/microorganisms9061205)
Supplement: Supplementary file 1 [file microorganisms-09-01205-s001.zip › 2020-06-01_Supp-Info-1_Chapter-3_New-Zealand.pdf]

Supplementary Information

An assessment of the *Lolium perenne* (perennial ryegrass) seedborne microbiome across cultivars, time, and biogeography: Implications for microbiome breeding

Methods S1 Endophyte Identification and Viability (KASP Assay)

Endophyte presence, identification, and viability were confirmed using a Kompetitive Allele-Specific PCR Assay (KASP). Seeds from each of the two cultivars (Alto and Trojan) containing different endophytes (AR1, AR37, SE, WE) were placed on filter paper dampened with tap water in petri dishes and assessed for germination over one week. Shoot samples (0.5 cm pieces) including the meristem were harvested for DNA extraction. A total of 44 replicates were extracted for cultivar-endophyte combinations Alto-AR1, Alto-AR37, and Trojan-AR37. A total of 22 replicates were extracted for cultivar-endophyte combinations Alto-WE and Alto-SE.

DNA extraction of seedlings was performed in 96-well plates using the QIAGEN MagAttract 96 DNA Plant Core Kit as per manufacturers' instructions with a minor modification. Modifications included the use of a Biomek FX liquid handling station. KASP primers were previously designed and generated by AgriBio and GeneWorks based on the allelic variation of single nucleotide polymorphisms (SNP) that differentiate endophytes from. The SNP allele-specific detection is based on a homogeneous, fluorescence assay, with each forward primer incorporating one distinct fluorescent dye (HEX, and FAM). Each reaction mixture (10.14 µL) consisted of 5 µL of 2x KASP master mix (GeneWorks), 0.14 µL of KASP assay mix (GeneWorks) and 5 µL of template DNA. The KASP PCR was performed in a Bio-Rad CFX96 (Bio-Rad) with the following program; a denaturation step at 94°C for 15 min; followed by 9 cycles of 94°C for 20 sec and 61°C for 60 sec (drop - 0.6°C / cycle); 26 cycles of 94°C 20 sec and 55°C for 60 sec; and a read step at 37°C for 60 sec. The data was visualised and analysed using Bio-Rad CFX manager 3.1 software to detect fluorescence and discriminate allelic variation between endophytes.

Methods S2 Illumina library preparation

The V4 region of the 16S rRNA gene was amplified using the following reagents: 12.5 µL 2X KAPA HiFi HotStart ReadyMix, 5µL of each of the 515F with adapter (5'-TCGTCGGCAGCGTCAGATGTGTATAAGAGACAG/GTGCCAGCMGCCGCGGTAA -3') and 806R (5'- GTCTCGTGGGCTCGGAGATGTGTATAAGAGACAG/GGACTACHVGGGTWTCTAAT -3') primers, 5 µL of 50 nM mP01 (GGCAAGTGTTCTTCGGA) and 5 µL of 50 nM pP01 (GGCTCAACCCTGGACAG) PNA

blockers, and 2.5 µL 5ng/µL of Template DNA to a final volume of 25 µL. The PCR reaction was run in an Agilent Surecylcer 8800 (Applied Biosystems) with the following conditions: denaturation at 95°C for 3 min; 25 cycles of 94°C for 30 sec, 75°C for 10 sec, 55°C for 10 sec, 72°C for 30 sec; and one final extension at 72°C for 5 min. The V4 region PCR amplicons were cleaned using AMPure XP beads. PCR amplicons were added to 20 µL of AMPure beads, vortexed for 2 min, incubated at room temperature, placed on to a magnet, washed twice with 80% ethanol, air dried for 10 minutes, and removed from the magnet. Amplicons (on AMPure beads) were added to 52.5 µL 10 mM Tris pH8.5, incubated for 2 mins, placed on to a magnet, and recovered in 50 µL of eluent. The purified V4 region PCR amplicons for each sample were indexed using the following reagents: 5 µL of the purified PCR amplicons, 5 µL Nextera XT Index Primer 1 (N7xx), 5 µL Nextera XT Index Primer 2 (S5xx), 25 µL 2X KAPA HiFi HotStart ReadyMix, and 10 µL sterile water. The PCR was run in an Agilent Surecylcer 8800 (Applied Biosystems) with the following conditions: denaturation at 95°C for 3 min; 8 cycles of 95°C for 30 sec, 55°C for 30 sec, and 72°C for 30 sec; and one final extension at 72° for 5 min. The PCR amplicon was purified using AMPure XP beads as described above with one modification; 27.5 µL 10 mM Tris pH8.5 was used as the final eluent.

Each of the indexed V4 region PCR amplicon libraries were quantified by NanoDrop and pooled to create equivalent concentrations. The pool was quantified by NanoDrop, Qubit, and TapeStation (Agilent) using high sensitivity DNA 1000 screening tapes.

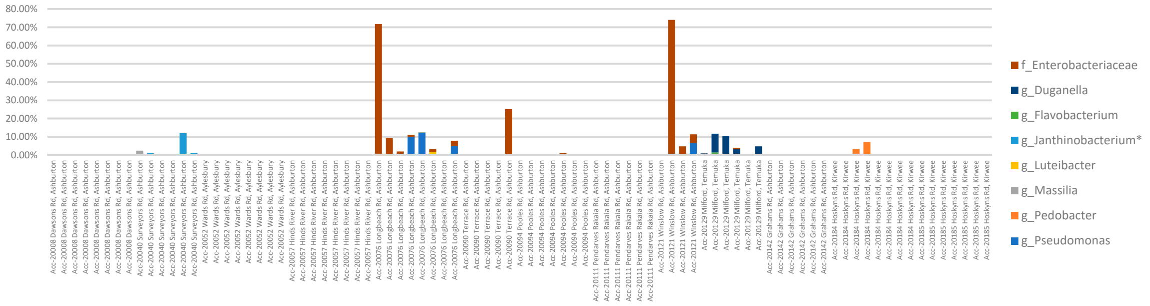

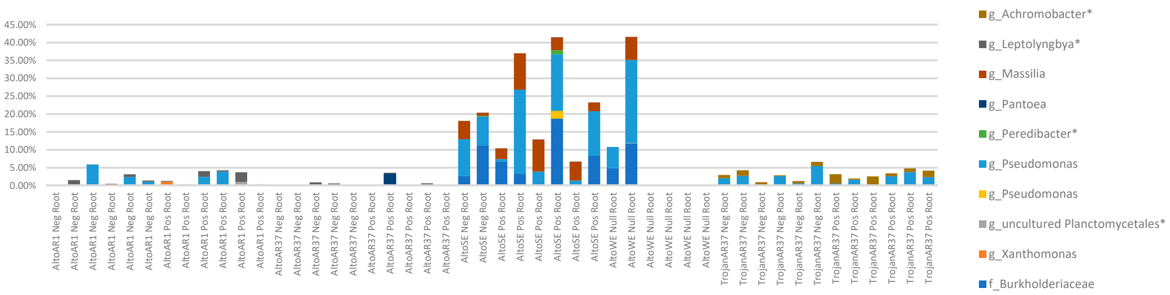

Figure S1 Comparison of the contribution of the 8 unique dominant taxa from Biogeography Seed (top) and 10 unique dominant taxa from Single Seed (bottom). Asterix in legends represents new taxa where there were no shared taxa from that Genus across both datasets.
